# Comparative Study of *Piper sylvaticum* Roxb. Leaves and Stems for Anxiolytic and Antioxidant Properties Through In Vivo, In Vitro, and In Silico Approaches

**DOI:** 10.3390/biomedicines8040068

**Published:** 2020-03-25

**Authors:** Md. Adnan, Md. Nazim Uddin Chy, A.T.M. Mostafa Kamal, Md Obyedul Kalam Azad, Kazi Asfak Ahmed Chowdhury, Mohammad Shah Hafez Kabir, Shaibal Das Gupta, Md. Ashiqur Rahman Chowdhury, Young Seok Lim, Dong Ha Cho

**Affiliations:** 1 Department of Bio-Health Technology, Kangwon National University, Chuncheon 24341, Korea; mdadnan1991.pharma@gmail.com (M.A.); azadokalam@gmail.com (M.O.K.A.); 2 Department of Pharmacy, International Islamic University Chittagong, Chittagong 4318, Bangladesh; nazim107282@gmail.com (M.N.U.C.); ashfak4u_ctg@yahoo.com (K.A.A.C.); mohammadshahhafezkabir@yahoo.com (M.S.H.K.); 3 Drug Discovery, GUSTO A Research Group, Chittagong 4000, Bangladesh; shaibaldasgupta88@gmail.com (S.D.G.); ashiq.ctgcu@gmail.com (M.A.R.C.); 4 Department of Chemistry, Wayne State University, Detroit, MI 48202, USA; 5 Department of Pharmacy, University of Science and Technology Chittagong, Chittagong 4202, Bangladesh; 6 Department of Chemistry, University of Chittagong, Chittagong 4331, Bangladesh

**Keywords:** *Piper sylvaticum*, anxiolytic, antioxidant, molecular docking, phytochemistry

## Abstract

*Piper sylvaticum* Roxb. is traditionally used by the indigenous people of tropical and subtropical countries like Bangladesh, India, and China for relieving the common cold or a variety of chronic diseases, such as asthma, chronic coughing, piles, rheumatic pain, headaches, wounds, tuberculosis, indigestion, and dyspepsia. This study tested anxiolytic and antioxidant activities by *in vivo*, *in vitro*, and *in silico* experiments for the metabolites extracted (methanol) from the leaves and stems of *P. sylvaticum* (MEPSL and MEPSS). During the anxiolytic evaluation analyzed by elevated plus maze and hole board tests, MEPSL and MEPSS (200 and 400 mg/kg, body weight) exhibited a significant and dose-dependent reduction of anxiety-like behavior in mice. Similarly, mice treated with MEPSL and MEPSS demonstrated dose-dependent increases in locomotion and CNS simulative effects in open field test. In addition, both extracts (MEPSL and MEPSS) also showed moderate antioxidant activities in DPPH scavenging and ferric reducing power assays compared to the standard, ascorbic acid. In parallel, previously isolated bioactive compounds from this plant were documented and subjected to a molecular docking study to correlate them with the pharmacological outcomes. The selected four major phytocompounds displayed favorable binding affinities to potassium channel and xanthine oxidoreductase enzyme targets in molecular docking experiments. Overall, *P. sylvaticum* is bioactive, as is evident through experimental and computational analysis. Further experiments are necessary to evaluate purified novel compounds for the clinical evaluation.

## 1. Introduction

Human neurological disarrays have instigated as ever-growing intimidation in the public health sector and significantly affected the function and quality of life [1]. Anxiety is a regular emotion but becomes appalling when it transpires too often and turns to a terrible psychiatric disorder [2]. Perhaps stress plays a vital part in the pathogenesis of anxiety. Moreover, the stressful state leads to oxidative stress, which has been described as a potential contributor to the pathogenesis of several chronic diseases, such as diabetes, liver damage, inflammation, aging, neurological disorders, and cancer [3,4]. To treat such chronic diseases, medicinal plants derived natural products have been used around the globe clinically, even for the management of normal fever to life-threatening conditions [5]. The rural people of Bangladesh consume medicinal plants as a primary source of health-care, so they play a pivotal role in treating a large number of diseases [6]. However, the folkloric practice of medicinal plants is mainly based on empirical shreds of evidence which need proper rationalization on scientific grounds.

*Piper sylvaticum* (Roxb.) belongs to the Piperaceae family; is a climbing herb, commonly known as pahari pipul (Hindi), pahaari peepal (Folk medicine), vana-pippali (Ayurveda), chang bing hu jiao (China), or the mountain long pepper (English). It is widely distributed in the tropical and subtropical countries such as India, Bangladesh, China, and Myanmar. The plant has several parts, such as leaves, the stem, roots, fruits, and seeds, and most of them have wide traditional uses for the treatment of various diseases such as rheumatic pain, headaches, chronic cough, cold, asthma, piles, diarrhea, wounds in lungs, tuberculosis, indigestion, dyspepsia, hepatomegaly, and pleenomegaly [7,8,9]. Besides, the root of this plant is used as carminative, and the aerial parts have diuretic actions [10]. The preliminary qualitative phytochemical analysis of this plant (leaves and stem) revealed the presence of several phytochemicals, including alkaloids, flavonoids, carbohydrates, tannins, and saponins. Additionally, an earlier quantitative phytochemical study of this plant reported that the plant contains substantial amounts of phenols (65.83 and 93.39 mg GAE/g dried extract), flavonoids (102.56 and 53.74 mg QE/g dried extract), and condensed tannins (89.32 and 55.82 mg CE/g dried extract) in the leaves and stem [9,11]. Besides, several phytoconstituents have been isolated from this plant, such as piperine, piperlonguminine, sylvamide, sylvatesmin, sylvatine, sylvone, piperic acid, sesamin, and beta-sitosterol; most of them are fall into the categories of alkaloids, alkamides, flavone, and lignins [9,12]. In addition, several pharmacological activities of this plant (leaf, stem, and root) have been reported previously. Kumar et al. reported antioxidant activity of the roots and fruits [13]. Paul et al. reported the anthelmintic activity of stem [14] and Haque et al. described the antidiarrheal activity of the stem [15]. Chy et al. stated that the plant (stem) has anti-nociceptive and anti-inflammatory properties [11]. Chy et al. also reported antibacterial, anthelmintic, and analgesic activities of the leaf part [9].

Even though the plant (*P. sylvaticum*) has numerous significant medicinal properties, hitherto, no studies have been performed to determine the anxiolytic and antioxidant activities of the leaf and stem parts. Therefore, this study aimed to investigate the anxiolytic and antioxidant activities of the methanol extracts of *P. sylvaticum* leaves and stems (MEPSL and MEPSS) in several experimental models, and an *in silico* molecular docking study was performed to identify the potential lead compounds of this plant for the aforementioned activity.

## 2. Materials and Methods

### 2.1. Drugs and Chemicals

Methanol, potassium ferricyanide, phosphate buffer, and ferric chloride (FeCl_3_), were obtained from Merck (Darmstadt, Germany). 1,1-diphenyl-2-picrylhydrazyl radical (DPPH) and trichloroacetic acid (TCA) were obtained from Sigma Chemicals Co. (St. Louis, MO, USA), and ascorbic acid from BDH Chemicals Ltd. (Poole, UK). Diazepam was obtained from Square Pharmaceuticals Ltd (Dhaka, Bangladesh). All other chemicals used in this study were of analytical reagent grade unless unless specified with an additional reference.

### 2.2. Plant Material Collection and Identification

The leaves and stems of *Piper sylvaticum* (Roxb.) were collected from Sita Pahar area of Kaptai, Rangamati district, Chittagong division (22°28′45”N 92°13′22”E and altitude: 14 m (49 feet), Bangladesh in October 2014, and the plant was identified by Dr. Shaikh Bokhtear Uddin, Taxonomist and Professor, Department of Botany, University of Chittagong. A voucher specimen number (SUB 3217) has been deposited at the Department of Pharmacy, International Islamic University, Chittagong, Bangladesh, and also in the Herbarium of the University of Chittagong for future reference.

### 2.3. Preparation of Extract

Approximately 400 g (leaves) and 220 g (stem) of the powdered materials were soaked in 700 and 900 mL of methanol, respectively at room temperature for 14 days with occasional stirring and shaking. Finally, the resultant mixture was filtered through a cotton plug, followed by Whatman No.1 filter paper (Sigma-Aldrich, St. Louis, MO, USA), and the filtrate solution evaporated to yield the methanol extract of *P. sylvaticum* leaves and stems (MEPSL and MEPSS). The detailed procedure was described in our previous articles—see materials and methods sections [9,11].

### 2.4. Experimental Animals and Ethical Statements

Swiss albino mice of both sexes (weighing about, 20–25 g) were collected from Jahangir Nagar University, Savar, Dhaka, Bangladesh. The animals were sheltered in polypropylene cages by maintaining suitable laboratory conditions (room temperature 25 ± 2 °C; relative humidity 55–60%; 12 h light/dark cycle) along with standard laboratory food and distilled water ad libitum. All the experimental works were conducted in noiseless conditions and the animals were acclimatized to laboratory conditions for 10 days before experimentation. This study was carried out in accordance with the internationally accepted principles for proper use of laboratory animal’s; namely, those of the National Institutes of Health (NIH) and the International Council for Laboratory Animal Science (ICLAS). The present study protocol was reviewed and approved by the “P&D committee” of the Department of Pharmacy, International Islamic University Chittagong, Bangladesh with a reference number: Pharm-P&D-61/08′16-125 (25/08/2016).

### 2.5. In vivo Study: Anxiolytic Activity

#### 2.5.1. Dosing Groups

In the present study, mice were randomly divided into six groups, and each group consisted of six mice (*n* = 6). Here, the control group received 1% Tween-80 in distilled water (Sigma-Aldrich, St. Louis, MO, USA); the positive control group received reference drug diazepam (1 mg/kg, body weight), whereas the remaining groups were given 200 and 400 mg/kg body weight of the MEPSL and MEPSS, individually.

#### 2.5.2. Elevated Plus Maze Test (EPM) in Mice

To prove the presence of anxiolytic compounds, a commonly known methodological tool is elevated plus maze (EPM), a rodent/experimental animal model which is used for the test [16]. With a height of 40 cm above from the ground, the design of the instrument is plus-shaped (+) with two opens arms (5 × 10 cm) and two closed arms (5 × 10 × l5 cm) diverging from a common point (5 × 5 cm). To avoid the occurrence of dropping down of the mice from the instrument, the open and the closed arms edges were kept 0.5 cm and 15 cm in height, respectively. After counting 30 min from the administration period of the test drug, each animal facing any of the enclosed arms was plotted in the middle of this instrument. Then total counting was noted in the open and closed entries for 5 min. When there was a sign of four paws in a single arm then that particular entry was recorded. The whole operation was conducted in a sound-proof room or equivalent by keeping eye on it from the nearby corner.

#### 2.5.3. Hole-Board Test in Mice

The hole board test is the widely used valid pharmacological method for assessing anxiolytic and/or anxiogenic activity [17]. The hole board apparatus consists of a wooden box (40 cm × 40 cm × 25 cm) with sixteen equidistant holes (diameter 3 cm) evenly distributed on the base of the box. The apparatus was elevated 25 cm above the floor. After 30 min of oral administration of treatments, each mouse was placed individually on the center of the board (facing away from the observer). Finally, the numbers of heads dipping in a period of 5 min were counted.

#### 2.5.4. Open Field Test in Mice

The spontaneous locomotor activities were assessed using the open field test [18]. Test animals were kept in the test room at least 1 h before each open field test for habituation. The apparatus comprised of a wood square box (50 cm × 50 cm × 40 cm) with the floor divided into twenty-five small squares of equal dimensions (10 cm × 10 cm) marked by black and white color. In this study, each test animal was placed individually at the center of the apparatus and observed for 5 min to record the number of squares crossed by the animal with its four paws. The open field arena was thoroughly cleaned by using isopropyl alcohol (70%) between each test to prevent each mouse from being influenced by the odors of urine and feces from the previous mouse.

### 2.6. In vitro Study: Antioxidant Activity

#### DPPH Free Radical Scavenging and Ferric Reducing Power Assays

The DPPH (1,1-diphenyl-1-picrylhydrazyl) free radical scavenging activities of the MEPSL and MEPSS were determined as described previously [19], and results were expressed as μg/mL in compared to reference standard ascorbic acid. Then, the reducing power assay of the both extract was determined based on the previously reported method [20,21], using ferric ion reducing antioxidant power, and the results were expressed as mean ± standard error mean (SEM). In both assays, change in absorbance was taken using UV–VIS Spectrophotometer (UVmini-1240, Shimadzu, Shimadzu Corporation, Kyoto, Japan).

### 2.7. Chemical Compounds Studied in this Article

Piperine (C_17_H_19_NO_3_), piperlonguminine (C_16_H_19_NO_3_), sylvamide (C_14_H_27_NO_3_), sylvatine (C_24_H_33_NO_3_), sylvatesmin (C_21_H_24_O_6_), and sylvone (C_23_H_28_O_8_) were selected through literature study, and the chemical structures of the compounds were downloaded from the PubChem compound repository: piperine (PubChem CID: 638024); piperlonguminine (PubChem CID: 5320621); sylvamide (PubChem CID: 21580215); sylvatine (PubChem CID: 90472536); sylvatesmin (PubChem CID: 3083590); and sylvone (PubChem CID: 15043005).

### 2.8. In silico Study: Molecular Docking Study

#### 2.8.1. Ligand and Protein Preparation

The chemical structures of six major compounds of *P. sylvaticum* were obtained from PubChem database (https://pubchem.ncbi.nlm.nih.gov/); then to prepare the ligand it was neutralized at pH 7.0 ± 2.0 and minimized by LigPrep tool (force field OPLS_2005) embedded in Schrödinger suite-Maestro version 10.1. Alternatively, three-dimensional crystallographic structures were retrieved from the Protein Data Bank RCSB PDB [22]: potassium channel (PDB: 4UUJ) [23] and xanthine oxidoreductase (PDB: 1R4U) [24]. These proteins were prepared for the docking experiment by Protein Preparation Wizard embedded in Schrödinger suite-Maestro version 10.1 (Schrödinger, LLC New York, NY, USA) as in the previously described method [5].

#### 2.8.2. Glide Standard Precision Docking Procedure

Molecular docking studies were performed to elucidate the possible mechanisms of the selected compounds against potassium channel and xanthine oxidoreductase receptors for anxiolytic and antioxidant activities. In this study, molecular docking experiments were carried out using Glide embedded in Maestro by standard precision scoring function, as in the previously described method [5].

### 2.9. Statistical Analysis

SPSS version 20 (Statistical package for the social sciences) software (Schrödinger, LLC New York, NY, USA) was used for data analysis and all comparisons were made by using one-way ANOVA followed by Dunnett’s test. Values were expressed as means ± SEM (standard errors of means) and standard deviations (SD), for which the *P*-values less than 0.05, 0.01, and 0.001 were considered statistically significant.

## 3. Results and Discussion

The present study was carried out to investigate the anxiolytic and antioxidant activities of the methanol extract of *P. sylvaticum* leaves and stem (MEPSL and MEPSS) through *in vivo*, *in vitro*, and *in silico* approaches. An earlier preliminary qualitative phytochemical study of this plant (both leaves and stem) reported that the plant contains numerous phytochemicals, such as alkaloids, flavonoids, carbohydrates, tannins, and saponins. Additionally, a quantitative phytochemical analysis of MEPSL also reported that it contains substantial amounts of phenol (65.83 mg gallic acid equivalent/g dried extract), flavonoids (102.56 mg quercetin equivalent/g dried extract), and condensed tannins (89.32 mg catechin equivalent/g dried extract). Furthermore, MEPSS contains significant amounts of phenol (93.39 mg), flavonoids (53.74 mg), and condensed tannins (55.82 mg) [9,11,14]. On the other hand, a previous acute toxicity study described that the plant had no mortality, abnormal behavior, and neurological changes up to 2000 mg/kg dose, which is a clear indication that the plant extract has low toxicity profile and is safe for a therapeutic dose [11].

Medicinal plants are the innumerable resources of pharmacologically active components. Plant-derived drugs have been demanding a very potential position due to their role as safer, cheaper, and effective drugs in the present world [25]. However, to develop a potential lead compound from a medicinal plant having multifarious pharmacological activities, various animal models and well-validated tests are inevitable in order to get a consistent preclinical and clinical decision [26]. In this study, we have presented a comparative pharmacological evaluation of *Piper sylvaticum*, to find whether leaves and stems of *P. sylvaticum* (MEPSL and MEPSS) have manifold pharmacological effects toward mitigating the anxiety disorder—the ultimate aim of our research. Then, we also explored the potentials of MEPSL and MEPSS for antioxidant activity, and finally a molecular docking study was performed to identify the possible lead compounds for the anxiolytic and antioxidant activity.

Both extracts of *P. sylvaticum* were evaluated for anxiolytic activity by employing the elevated plus maze (EPM) animal model, which is very popular due to the rapid assessment of the anxiety modifying reactions in mice [27]. The typical EPM tool has two opposite open and two bounded arms, whereas the open arena is thought to be more abysmal for the animals, and an anxiolytic agent can motivate the mice toward open arm exploration [28]. Table 1 demonstrated the anxiolytic activity of MEPSL and MEPSS in the EPM test. Administration of MEPSL and MEPSS (400 and 200 mg/kg, body weight) revealed a dose dependent increase in locomotion. Particularly, 400 mg/kg significantly (*P* < 0.01) elevated the amount of time spent in the open arms. Among both extracts, MEPSL was very effective, and 400 mg/kg remarkably enhanced spending time (109.65 ± 4.88) (*P* < 0.01) and the number of entries (11.33 ± 1.33) (*P* < 0.001) in the open arms. Similarly, 200 mg/kg showed a moderate (82.73 ± 3.03) but significant (*P* < 0.01) anxiolytic effect compared to the control group (78.50 ± 4.75). In addition, reference drug (diazepam at 1 mg/kg, i.p.) treated mice exposed an obvious provocation in the time spent and number of entries in the open arms.

In the same way, with the hole board test (HBT) we intended to determine the exploratory responses as well as numerous extents of the undefined behavior of a mouse to an unacquainted atmosphere [29]. The demonstration of hole poking (head dipping) inclination specifies a high level of anxiolytic activity, while reluctance of the hole visiting indicates high level of anxiety [30]. In this test, mice treated with MEPSL and MEPSS (200 and 400 mg/kg, body weight) displayed noteworthy exploratory behavior in a dose dependent way (Table 2). The treatment of 400 mg/kg exposed significant hole poking tendencies for both extracts; a higher number of head dips resulted with 200 mg/kg. In addition, the positive control diazepam (1 mg/kg, i.p.) also manifested more head dipping compared to the control group.

Further we confirmed the possibility of locomotor and exploratory activity of MEPSL and MEPSS through open field assay. The conditions of this test were highly anxiogenic, for which most standard anxiolytic agents are identified from this assessment [31]. In our study, dose (MEPSL and MEPSS at 200 and 400 mg/kg, body weight) administration significantly stimulated locomotion and exploration tendency in mice (Table 3). In this test, the lower dose (200 mg/kg) exhibited maximum agility and CNS (central nervous system) exciting effects, while exploration and locomotion of MEPSL at both doses were almost identical at all intervals over 120 min. It was reported that anxiolytics with low doses improved the anxiety state by altering motor activity followed by suppressing the muscle relaxation [31]. In contrast, the reference drug (diazepam, 1 mg/kg) produced quietness or CNS depressant-like activity. Importantly, CNS depressant-drug-like benzodiazepines inhibit excitation and curiosity in mice against the new ambient which decreases their locomotion tendency in consequence [32]. The neurobiological mechanism of anxiety is the result of either an imbalance of neurotransmitter (dopamine, GABA, and serotonin) function or dysregulation of glutamatergic, serotonergic, GABA-ergic, and noradrenergic transmission [33]. In our experiment, extracts of *P. sylvaticum* may exert anxiolytic actions by modifying neurotransmitter synthesis and functions. It is supposed that active components of *P. sylvaticum* interact with the neurotransmitter or neuromodulator receptors, which regulate the neuronal communication, stimulate the CNS activity, and improve the function of endocrine systems [34]. Additionally, it has been previously reported that the plant contains flavonoids, saponins, and tannins that are responsible for the anxiolytic activity [35], and our earlier qualitative and quantitative phytochemical studies revealed that the plant contains alkaloids, flavonoids, phenol, tannins, and saponins [9,11]. Thus, the anxiolytic activities of the MEPSL and MEPSS might be due to the binding of any of these phytochemicals to the GABAA-BZDs complex.

**Figure 1:**
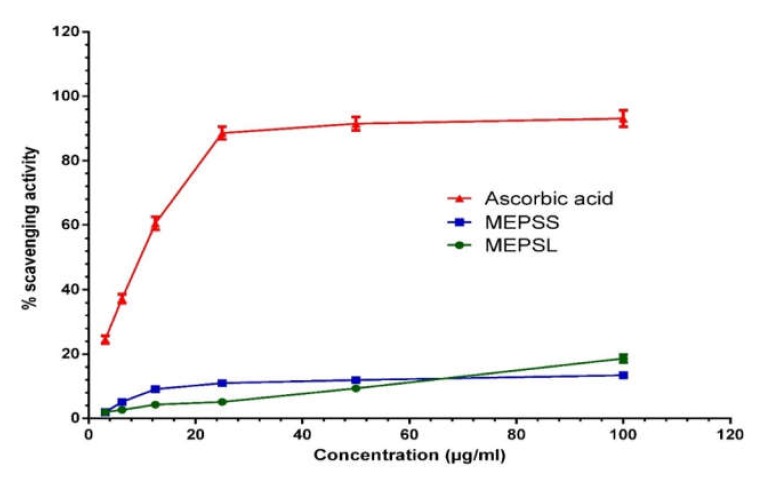
DPPH free radical scavenging activities of MEPSL and MEPSS compared with the reference standard ascorbic acid. Percentage of DPPH free radical scavenging activity by different concentrations of the MEPSL, MEPSS, and reference standard ascorbic acid. Values are expressed as mean ± SD (*n* = 3). MEPSL, methanol extract of *Piper sylvaticum* leaves; MEPSS, methanol extract of *Piper sylvaticum* stem.

Stressful conditions, such as EPM, HC, and OP tests, can boost up the production of reactive oxygen species (ROS) in mice and prevails over their brain defenses. However, the interplay relationship between oxidative stress (OS) and neurological disorders is not surprising [36]. *Rammal H* et al. 2008 revealed a clear interlink between anxiety and OS wherein such an imbalance of the redox system in mice led to the development of neuro-degeneration and chronic inflammation [37]. In this regard, antioxidant therapy may improve the neuronal functions and OS by inhibiting ROS formation. As shown in Figure 1, free radical scavenging capacity of MEPSL and MEPSS were concentration dependent, wherein the highest DPPH scavenging capacity was observed for MEPSL at highest concentration (100 μg/mL). In addition, 50% inhibitory concentration (IC_50_) values were found 288.39 μg/mL for MEPSL and 476.97 μg/mL for MEPSS, respectively, while ascorbic acid showed 6.87 μg/mL.

**Figure 2:**
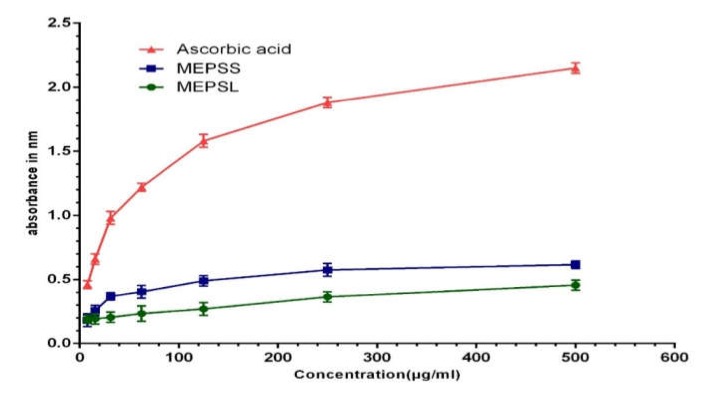
Reducing power capacity of MEPSL and MEPSS compared with the reference standard ascorbic acid. Values are expressed as mean ± SEM (*n* = 3). MEPSL, methanol extract of *Piper sylvaticum* leaves; MEPSS, methanol extract of *Piper sylvaticum* stem.

To reassess the antioxidant ability of MEPSL and MEPSS in reducing Fe^3+^ to Fe^2+^ ions, we conducted ferric reducing antioxidant power assay (FRAP). Both extracts were found as strong antioxidant, confirmed by color change from yellow (test solution) to green and prussian blue, indicating reduction of Fe^3+^ to Fe^2+^ ions. The reduction was also monitored by UV-vis analysis at 700 nm, as increased absorbance is proportional to higher reduction of Fe^3+^ ions [38]. Data for the reducing power of MEPSL and MEPSS was shown in Figure 2, and a dose-dependent reducing capability was observed compared to reference standard ascorbic acid. Scientific investigations have been reported previously that phenolic compounds are responsible for the free radical scavenging effect of the plant and also could play an essential role in the reducing power of the plant extract [39,40,41]. An earlier quantitative phytochemical study of this plant revealed that it contains a considerable amount of polyphenols such as flavonoids, phenols, and condensed tannins [9,11]. Thus, it might be possible that the presence of such phytochemicals could be responsible for the free radical scavenging activity and ferric reducing power capacity of both extract.

The previous phytochemical study revealed the presence of various phytochemicals in the MEPSL and MEPSS like alkaloids, flavonoids, carbohydrates, tannins, and saponins. In addition, an earlier quantitative phytochemical analysis of this plant indicated the highest amount of polyphenols contents in the both plant extract [9,11]. Furthermore, piperine, piperlonguminine, sylvamide, sylvatine, sylvatesmin, and sylvone were selected based on the availability as major compounds through literature review where most of them are fall in the categories of flavones, lignans, amide alkaloids, and alkaloids [9,12]. After the selection of compounds, an *in silico* molecular docking study was performed. Molecular docking is a key tool which has been widely used for the drug development process. It is a form of structure-based process that measures the binding affinities between small molecules and macromolecular targets like proteins. Moreover, it is also used to understand the possible molecular mechanism of action of various pharmacological responses [5,42]. From this understanding, this study was performed to comprehend the molecular mechanism of action better and to correlate their findings with the experimental results. In the present study, six major selected phytocompounds of *P. sylvaticum* were docked against two target enzyme or receptor, viz. the potassium channel receptor (PDB: 4UUJ), and xanthine oxidoreductase (PDB ID: 1R4U) enzyme, and the docking scores obtained for all compounds have been reported in Table 4.

**Figure 3:**
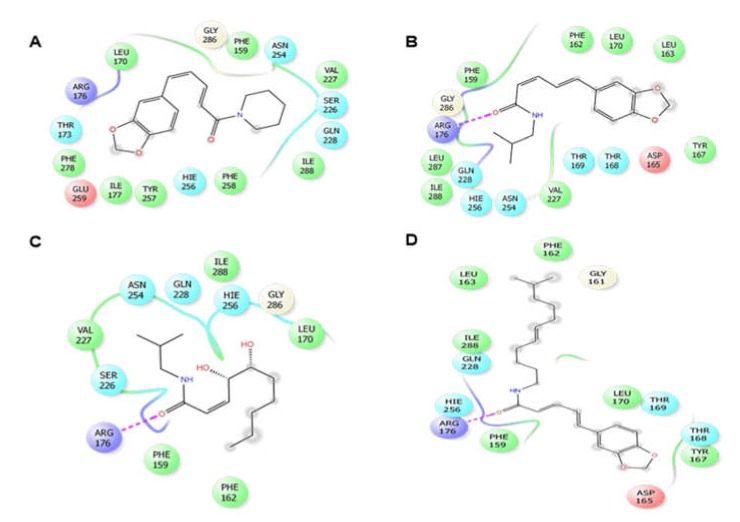
2D interactions of the piperine (**A**), piperlonguminine (**B**), sylvamide (**C**), and sylvatine (**D**) with the active site of xanthine oxidoreductase (PDB: 1R4U). Colors indicate the residue (or species) type: Red—acidic (Asp, Glu), green—hydrophobic (Ala, Val, Ile, Leu, Tyr, Phe, Trp, Met, Cys, Pro), purple—basic (Hip, Lys, Arg), blue—polar (Ser, Thr, Gln, Asn, His, Hie, Hid), light gray—other (Gly, water), and darker gray—metal atoms. Interactions with the protein are marked with lines between ligand atoms and protein residues: Solid pink: H—bonds to the protein backbone, Dotted pink: H-bonds to protein side chains, Green: pi-pi stacking interactions, Orange: pi-cation interactions. Ligand atoms exposed to solvent are marked with gray spheres. The protein “pocket” is displayed with a line around the ligand, colored with the color of the nearest protein residue. The gap in the line shows the opening of the pocket.

**Figure 4:**
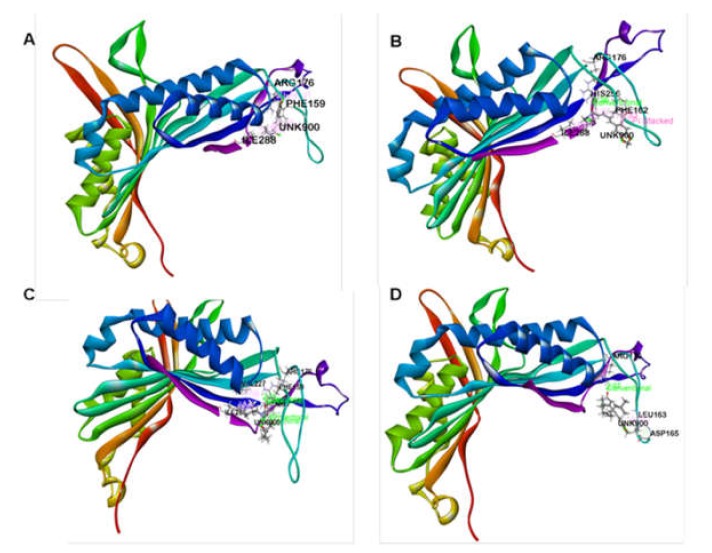
Best ranked pose of piperine (**A**), piperlonguminine (**B**), sylvamide (**C**), and sylvatine (**D**) in the binding pocket of xanthine oxidoreductase (PDB: 1R4U).

In the case of antioxidant docking study, the six selected phytocompounds of *P. sylvaticum* were docked against xanthine oxidoreductase (PDB ID: 1R4U) and showed docking scores ranging from +1.33 to −3.43 kcal/mol. The result of the docking study is shown in Table 4, and the docking figure is presented in Figure 3 and Figure 4. From the results, it is clear that the phytocompounds piperine (−3.43 kcal/mol) displayed the highest scores against target enzyme, followed by piperlonguminine (−2.65 kcal/mol), sylvatine (−1.15 kcal/mol), and sylvamide (+1.33 kcal/mol). Here, piperine interacts with the target enzyme through two pi-alkyl interactions with Phe159 and Arg176, and one alkyl interaction with Ile288. Piperlonguminine interacted with the same enzyme by forming one hydrogen bond with Arg176, one alkyl bond with Ile288, one pi-alkyl bond with His256, and one pi-pi stacked with Phe162. Sylvatine showed the interactions forming two hydrogen bond with Arg176 and Asp165, and one alkyl interaction with Leu163 while sylvamide showed five hydrogen bond interactions with Arg176 (two interactions), His256 (two interactions), and Ile288, and three hydrophobic interactions with Val227, Ile288, and Phe159. The reference drug, ascorbic acid showed seven hydrogen bond interactions with Arg176 (two interactions), Val227, His256 (two interactions), Asn254, and Gln228. However, sylvatesmin and sylvone did not dock with the target receptor/enzyme at all.

**Figure 5:**
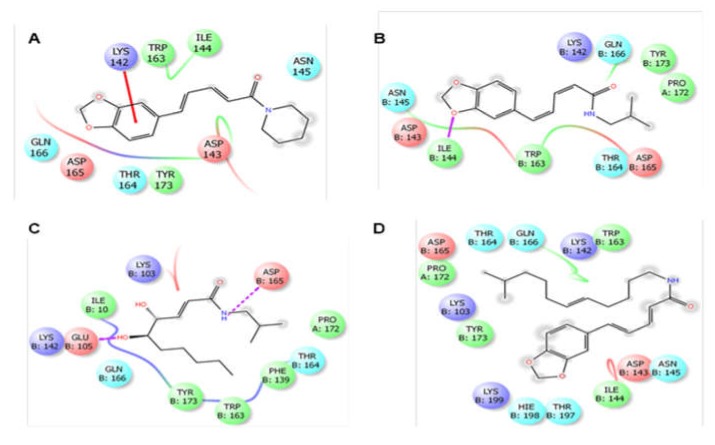
2D interactions of the piperine (**A**), piperlonguminine (**B**), sylvamide (**C**), and sylvatine (**D**) with the active site of potassium channel (PDB: 4UUJ).

**Figure 6:**
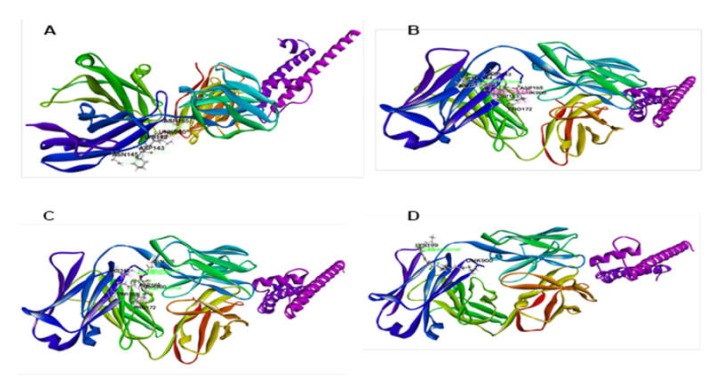
Best ranked pose of piperine (**A**), piperlonguminine (**B**), sylvamide (**C**), and sylvatine (**D**) in the binding pocket of potassium channel (PDB: 4UUJ).

In the case of anxiolytic docking study, results are shown in Table 4, and the most representative interactions between ligands and receptors have been presented in Figure 5 and Figure 6. Our study showed that piperine and sylvamide have shown the highest and lowest binding affinities against the potassium channel (PDB: 4UUJ) with docking scores of −4.12 kcal/mol and −0.59 kcal/mol respectively. The ranking order of docking score for anxiolytic effect is given below: piperine > piperlonguminine > sylvatine > sylvamide.

**Figure 7:**
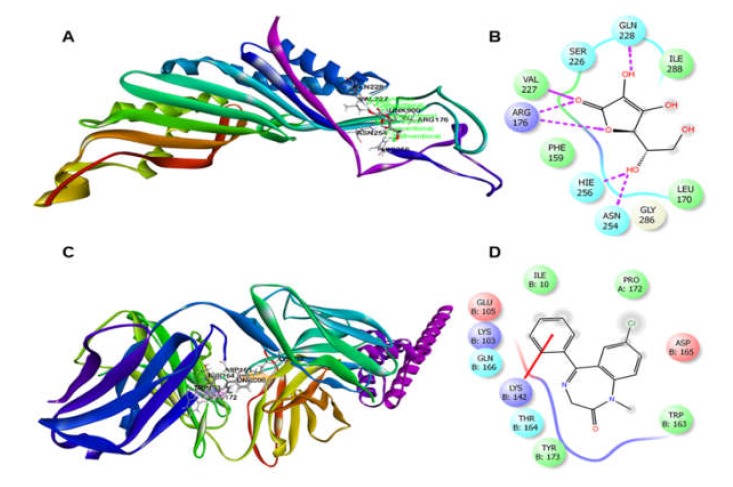
(**A**) Best ranked pose and (**B**) 2D interaction diagram of ascorbic acid docked at the binding pocket of xanthine oxidoreductase (PDB: 1R4U). (**C**) Best ranked pose and (**D**) 2D interaction diagram of diazepam docked at the binding pocket of potassium channel (PDB: 4UUJ).

The molecular docking study of each compound displayed several binding interactions between the ligands and the target receptor. Here, piperine interacts with the potassium channel (PDB: 4UUJ) receptor through five hydrogen bonds to Asp165 (two interactions), Asn145 (two interactions), Asp143, and one pi-alkyl interaction with Lys142. Piperlonguminine interacted with the same receptor by forming five hydrogen bonds to Ile144 (two interactions), Asp143, Asp165, and Asn145; two pi-pi stacked interactions with Trp163; and one alkyl interaction with Pro172, whereas sylvatine showed only one hydrogen bond interaction with Lys199. Besides, sylvamide interacted with the same receptor by forming four hydrogen bonds to Lys142, Glu105, and Asp165 (two interactions); three alkyl interactions with Pro172 (two interactions) and Lys142; and one pi-alkyl interaction with Trp163. The standard drug (Table 4 and Figure 7), diazepam showed one hydrogen bond interaction with Thr164 and five hydrophobic interactions with Lys103, Asp165, Trp163 (two interactions), and Pro172. However, sylvatesmin and sylvone did not dock with the target receptor/enzyme.

From these results, we can conclude that the studied phytocompounds, particularly piperine, piperlonguminine, sylvamide, and sylvatine, may in part be responsible for the anxiolytic and antioxidant activities of the plant extract through interactions with these target enzyme or receptor. It has been previously reported that piperine has anxiolytic [43] and antioxidant [44] activities. Another phytoconstituent, piperlonguminine, has been reported to have multifarious pharmacological potentials, including anticancer, analgesic, cytotoxic, antioxidant, anxiolytic, and antidepressant ones [45,46]. In addition, sylvamide possesses a wide range of pharmacological effects, such as antimicrobial, neuropharmacological, antioxidant, anxiolytic, and hepatoprotective ones. This compound is also used to treat cognitive disorders [47].

## 4. Conclusions

In summary, results of the present study revealed that both extracts (MEPSL and MEPSS) possesses significant anxiolytic and antioxidant activities. These activities might be due to the presence of high polyphenol content in both extracts and could be due to the individual or synergistic effects of different phytochemicals, such as alkaloids, flavonoids, saponins, tannins, and phenols. Additionally, our molecular docking study unveiled that piperine, piperlonguminine, sylvamide, and sylvatine have higher binding affinities towards the target receptor/enzymes for anxiolytic and antioxidant activity, respectively. It might be possible that, for these four phytocompounds responsible for the observed pharmacological responses, further study is still necessary to elucidate their in-depth molecular mechanisms of action in animal models.

## Figures and Tables

**Table 1:** Anxiolytic effects of MEPSL, MEPSS, and diazepam on behavior of mice in elevated plus-maze model test in mice.

Treatment (mg/kg)	Time Spent in Open Arm (sec)	No. of Entry in Open Arm
Control	78.50 ± 4.75	9.50 ± 1.96
RSD 1	119.16 ± 5.45	13.83 ± 1.25 *
MEPSL 200	82.73 ± 3.03 **	9.66 ± 1.42 *
MEPSS 200	69.93 ± 3.65	6.83 ± 1.60 *
MEPSL 400	109.65 ± 4.88 **	11.33 ± 1.33 ***
MEPSS 400	93.89 ± 3.66 **	7.33 ± 1.49

Each value is expressed as mean ± SEM (*n* = 6). * *P* < 0.05, ** *P* < 0.01, and *** *P* < 0.001 compared with the control group (Dunnett’s test). MEPSL, methanol extract of *Piper sylvaticum* leaves; MEPSS, methanol extract of *Piper sylvaticum* stem; RSD: reference standard drug (Diazepam, 1 mg/kg).

**Table 2:** Anxiolytic effects of MEPSL, MEPSS, and diazepam in hole board test in mice.

Treatment (mg/kg)	No. of Head Dipping	Latency to the First Head Dipping (sec)
Control	31.16 ± 3.12	20.83 ± 1.52
RSD 1	67.66 ± 1.90	2.46 ± 0.42 **
MEPSL 200	41.16 ± 2.53 ***	8.91 ± 0.16 **
MEPSS 200	37.33 ± 2.10 *	6.95 ± 1.07
MEPSL 400	56.16 ± 4.70 *	5.28 ± 0.27 ***
MEPSS 400	49.83 ± 3.98 **	3.35 ± 1.02 **

Each value is expressed as mean ± SEM (*n* = 6). *P < 0.05, ** *P* < 0.01, and *** *P* < 0.001 compared with the control group (Dunnett’s test). MEPSL, methanol extract of *Piper sylvaticum* leaves; MEPSS, methanol extract of *Piper sylvaticum* stem; RSD: reference standard drug (Diazepam, 1 mg/kg).

**Table 3:** Anxiolytic effects of MEPSL, MEPSS, and diazepam on a number of movements in open field test in mice.

Treatment (mg/kg)	No. of movements				
	0 min	30 min	60 min	90 min	120 min
Control	86.16 ± 4.59	71.83 ± 3.82	67.66 ± 4.25	60.50 ± 3.58	54.83 ± 5.23
RSD 1	79.16 ± 5.32	46.83 ± 4.26 **	35.66 ± 4.21	17.50 ± 3.25*	11.33 ± 2.36 ***
MEPSL 200	76.50 ± 2.48	58.83 ±6.56*	43.83 ±7.40	37.66 ±7.05*	28.83 ± 2.32 *
MEPSS 200	85.50 ± 6.69	51.16 ± 5.52	34.83 ± 5.90 **	22.33 ± 5.01	18.33 ± 1.45 **
MEPSL 400	91.50 ± 2.01	63.83 ± 2.93 ***	48.83 ± 2.49	36.33 ± 1.72 *	32.16 ± 1.47 **
MEPSS 400	79.33 ± 6.57	56.83 ± 3.44*	38.83 ± 3.74 **	31.66 ±2.23 **	20.16 ± 5.61

Each value is expressed as mean ± SEM (*n* = 6). * *P* < 0.05, ** *P* < 0.01, and *** *P* < 0.001 compared with the control group (Dunnett’s test). MEPSL, methanol extract of *Piper sylvaticum* leaves; MEPSS, methanol extract of *Piper sylvaticum* stem; RSD: reference standard drug (Diazepam, 1 mg/kg).

**Table 4:** Docking scores and binding interactions of the selected compounds with xanthine oxidoreductase (PDB: 1R4U) and potassium channel (PDB: 4UUJ) for antioxidant and anxiolytic activity respectively.

Compounds	Docking score (kcal/mol)	Hydrogen bond interactions		Hydrophobic interactions	
		Amino acid residue	Distance (Å)	Amino acid residue (bond)	Distance (Å)
Antioxidant activity: Xanthine oxidoreductase (PDB: 1R4U)					
Piperine	−3.43	-	-	Ile288 (Alkyl)	5.09
				Phe159 (Pi-Alkyl)	4.95
				Arg176 (Pi-Alkyl)	5.46
Piperlonguminine	−2.65	Arg176	2.35	Phe162 (Pi-Pi Stacked)	5.83
				Ile288 (Alkyl)	4.48
				His256 (Pi-Alkyl)	4.93
Sylvamide	+1.33	Arg176	2.03	Val227 (Alkyl)	3.89
		Arg176	2.60	Ile288 (Alkyl)	4.43
		His256	2.86	Phe159 (Pi-Alkyl)	5.22
		His256	2.87		
		His256	2.75		
		Ile288	3.59		
Sylvatine	−1.15	Arg176	2.06	Leu163 (Alkyl)	4.88
		Asp165	2.20		
Sylvatesmin	-	-	-	-	-
Sylvone	-	-	-	-	-
Ascorbic acid (standard)	−5.13	Arg176	2.34	-	-
		Arg176	2.16		
		Val227	2.14		
		His256	2.07		
		His256	2.56		
		Asn254	1.99		
		Gln228	2.13		
**Anxiolytic activity: Potassium channel (PDB: 4UUJ)**					
Piperine	−4.12	Asp165	2.45	Lys142 (Pi-Alkyl)	4.11
		Asp143	2.82		
		Asn145	2.38		
		Asn145	2.75		
		Asp165	3.02		
Piperlonguminine	−4.03	Ile144	2.34	Trp163 (Pi-Pi Stacked)	4.41
		Asp143	2.57	Trp163 (Pi-Pi Stacked)	5.20
		Asp165	2.63	Pro172 (Alkyl)	5.08
		Ile144	2.48		
		Asn145	2.68		
Sylvamide	−0.59	Lys142	2.31	Lys142 (Alkyl)	3.88
		Glu105	1.91	Pro172 (Alkyl)	4.96
		Asp165	1.84	Pro172 (Alkyl)	4.27
		Asp165	2.54	Trp163 (Pi-Alkyl)	4.13
Sylvatine	−2.53	Lys199	2.88	-	-
Sylvatesmin	-	-	-	-	-
Sylvone	-	-	-	-	-
Diazepam (standard)	-3.81	Thr164	2.50	Lys103 (Pi-Cation)	4.86
				Asp165 (Pi-Anion)	4.50
				Trp163 (Pi-Sigma)	2.76
				Pro172 (Alkyl)	5.02
				Trp163 (Pi-Alkyl)	4.13

## Abbreviations

MEPSL	Methanol extract of *Piper sylvaticum* leaves
MEPSS	Methanol extract of *Piper sylvaticum* stem
PDB	Protein data bank
OPLS RMSD SPSS SEM BZDs GAE QE CE	Optimized potentials for liquid simulations Root-mean-square deviation Statistical package for the social sciences Standard error of the mean Benzodiazepines Gallic acid equivalent Quercetin equivalent Catechin equivalent
